# Objectively Measured Sedentary Time in Children and Their Parents

**DOI:** 10.3934/publichealth.2016.4.823

**Published:** 2016-09-30

**Authors:** Adrienne R Hughes, David J Muggeridge, Ann-Marie Gibson, Avril Johnstone, Alison Kirk

**Affiliations:** 1 Physical Activity and Health group, School of Psychological Science and Health, Graham Hills Building, 40 George Street, University of Strathclyde, Glasgow, UK; 2 Institute of Clinical Exercise and Health Science, School of Science and Sport, University of the West of Scotland, Hamilton, UK

**Keywords:** Sedentary behaviour, objective measurement, children, adolescents, activPAL

## Abstract

**Background:**

No studies have examined associations in objectively measured sedentary time between parents and young people using activPAL posture sensors, which provide a more accurate estimate of sedentary time compared to accelerometer-based devices. This study examines patterns and associations of activPAL measured sedentary time and number of sedentary breaks on weekdays and weekend days in preschool (2–4 yrs), primary (5–11 yrs) and secondary school aged children (12–17 yrs) and their parents.

**Methods:**

51 parents (16 M, 35 F; mean age 39 (+/−8) yrs) and 51 children (28 M, 23 F; mean age 9 (+/−5) yrs) wore an activPAL monitor for 7 days to measure time spent sedentary and number of breaks in sedentary time. Data was assessed by Pearson's correlations and t-tests.

**Results:**

Secondary school children spent a greater percentage of their day sedentary (64.5 (+/−8.5) %) than preschool (57.4 (+/−7.3) %) and primary school children (57.2 (+/−5) %). For the secondary school parent dyad, there were no significant positive associations for time sedentary (*r* = −0.167, *p* = 0.494) and percentage of day sedentary (*r* = −0.247, *p* = 0.308). For the primary school parent dyad, there were medium, but non-significant positive correlations for time sedentary (*r* = 0.38, *p* = 0.146) and percentage of day sedentary (*r* = 0.363, *p* = 0.167). For the preschool parent dyad, there were medium—large positive correlations for percentage of waking day sedentary at weekends (*r* = 0.479, *p* = 0.083) and number of sedentary breaks (*r* = 0.648, *p* = 0.012) at weekends.

**Conclusions:**

There were positive associations in sedentary time between primary school children and their parents, and between preschool children and their parents at the weekend. Thus, interventions aimed at reducing sedentary time of parents and children together, particularly at the weekend for young children, may be effective in these age groups. Secondary school children were more sedentary and had fewer sedentary breaks than younger children, thus interventions should promote breaks in sedentary time as well as reducing total sedentary time in this age group.

## 1. Introduction

Sedentary behaviours are defined as waking behaviours that require low energy expenditure (≤1.5 metabolic equivalents) and are performed in a sitting or reclining posture [1]. Key sedentary behaviours in young people include sitting at screens (e.g. TVs, tablets, smartphones, computers), for transport and at school [1]. Evidence shows that young people spend a large proportion of their waking day engaging in sedentary behaviours, and that sedentary time (defined as total time spent in sedentary behaviours during waking hours) increases with age across childhood and adolescence [2]–[4]. A recent study used accelerometers to measure sedentary time and questionnaires to measure screen time in 9–11 year old children. Data were collected from 12 sites around the world and found that, on average, children spent 8.6 hours/day being sedentary and 54% of children exceeded the 2 hours of recommended screen time a day [3].

Research suggests that these high levels of sedentary time in young people may have adverse effects on physical and psychosocial health [5], though most of the current evidence is based on cross-sectional studies and assessment of screen time (which does not adequately represent total sedentary time [6]–[8]. Nevertheless, there is strong evidence that high levels of sedentary time are associated with negative health outcomes in adults [9],[10], and that sedentary time tracks from childhood to adulthood [11]. It therefore seems apparent that there is a need for interventions to reduce sedentary time in young people.

It is important to identify the key correlates of sedentary time in young people so that these correlates can be targeted in interventions to reduce sedentary time [12]. It is also likely that the correlates of sedentary time vary by age, time of day and day of the week (i.e. weekdays vs weekends), and may differ for screen/TV viewing and total sedentary time, thus requiring different intervention strategies to reduce sedentary behaviour [12]. It is generally thought that a child's behaviour is heavily influenced by their parent's behaviour, therefore parental sedentary time may be a key correlate of sedentary time in children and adolescents and an important target for intervention [12]. Several mechanisms may explain potential parent-child associations in sedentary time. Parents who engage in high levels of sedentary time themselves may be more likely to engage in non-screen-based and screen-based sedentary behaviours with their children (e.g. watching TV together, driving their child to and from school and other locations rather than walking, reading, drawing or playing sedentary games together), or may be less likely to (1) set limits on the amount of time their child engages in non-screen-based and screen-based sedentary behaviours; or (2) encourage more active behaviours or (3) provide home environments that limit sedentary behaviours or a combination of these [12].

Whilst collectively investigating the behaviours of children and their parents appears important, research is also warranted to investigate this at a sub level. For example, the National Health and Nutrition Examination Survey (NHANES) reported ∼6.1, 7.5 and 8.0 h/day of sedentary time in children aged 6–11, 12–15 and 16–19 years old, respectively [13]. These data suggest that a child's behaviour may alter as they mature, however it is unclear whether the behaviour is linked to that of their parents or not. It is possible that as children get older they spend less time with their parents and therefore may be less likely to be influenced by their parents' sedentary time. It therefore appears apparent that research utilising child groups of various ages would be beneficial and help understand the relationship between children and their parents further.

Several studies have explored the association for self and proxy-reported measures of screen time/TV viewing between parents and children [14]–[22]. However, evidence shows that self- and proxy-reported screen time/TV viewing can be inaccurate [23] and is not a good marker of total sedentary time in children [6] or adults [24]. In contrast, few studies have explored the association between parent and child total sedentary time using objective methods, and even the studies that have explored parent-child associations using objectively measured total sedentary time in children, have tended to use self-reported screen time/sedentary time for parents [25]–[29]. Nevertheless, a small number of studies have examined parent-child associations using accelerometer-based motion sensors to measure sedentary time in children and their parents but have reported mixed associations [30]–[32]. In addition, accelerometer-based motion sensors determine sedentary time based on a lack of movement under a specified accelerometer cut point, therefore standing still may be mis-classified as sitting/lying, leading to an overestimation of sedentary time [33]–[34]. In contrast, the activPAL (a posture sensor) uses an inclinometer to detect posture which means it can differentiate between sitting/lying and standing, and is therefore more accurate at estimating sedentary time [33]. A recent study by De Decker et al [34] found that sedentary time measured with the ActiGraph was 7.7% higher than sedentary time measured with the activPAL in preschool children, and concluded that the difference in sedentary time was mainly due to the inclusion of standing in the ActiGraph output.

To our knowledge, no studies have examined associations in objectively measured sedentary time between children and adolescents and their parents using activPAL posture sensors, which provide a more accurate estimate of sedentary time compared to accelerometer-based devices. Furthermore, there is evidence to suggest that prolonged periods of continuous sedentary behaviour are harmful to health, whereas frequent breaks in sedentary behaviour can help to counteract some of these harmful effects [35]. To our knowledge, no studies have used the activPAL device to measure frequency of sedentary breaks in children and adolescents or examined the association in frequency of sedentary breaks between parents and young people. Therefore, the primary aim of this study was to examine associations between parent and child activPAL measured sedentary time and number of breaks in sedentary time on weekdays and weekend days across three age groups of children (i.e. pre-school children age 2–4 years, primary school children aged 5–11 years and adolescents aged 12–17 years).

## 2. Methods

### 2.1. Participants

Parents (aged between 18 and 65 years) who had children between the ages of 2 and 17 years old were recruited from a University campus in Glasgow and surrounding areas using posters, email alerts and word of mouth from January to March 2014. A total of 51 parent and child dyads were recruited and subsequently categorised, based on the child's age, into one of three groups: preschool children aged 2–4 years who were walking unaided and not attending school; primary school children aged 5–11 years; secondary school children aged 12–17 years. Parents received an information sheet, and provided written informed consent for themselves and their child prior to participation. Ethical approval was granted by the School of Psychological Sciences and Health ethics committee at the University of Strathclyde.

### 2.2. Procedures

Parents who provided informed consent were contacted to arrange a meeting at their home or an appropriate alternative location. At this meeting the parent was asked to complete a demographic questionnaire to self report the height and body mass of themselves and that of their child to the nearest 1 cm/1 kg respectively. Body mass index (BMI) of parents and children was calculated using the equation: BMI = weight (kg)/height (m)^2^. Each parent and child was fitted with an activPAL monitor (PAL Technologies Ltd., Glasgow, UK), which was taped onto the frontal thigh of the left leg in accordance with standard procedures. The activPAL was made waterproof by wrapping the device in an adhesive covering in order to increase retention by not needing to remove during washing/swimming. Parents and children were asked to maintain their normal lifestyles and wear the activPAL for 24 hours/day for 7 consecutive days. Participants were also given a diary to record the time they woke up and went to sleep each day, and if the activPAL was removed and reasons for removal. After one week, parents returned the activPALs and diaries in person to the research team.

### 2.3. Measurement of Sedentary Time and Breaks in Sedentary Time

The activPAL is a small lightweight device, worn on the thigh, which measures posture allocation and classifies an individual's free living activity into time spent sitting/lying, standing and stepping [32]. The device differentiates between sitting/lying and upright posture by determining the position of the thigh using an inclinometer and also measures movement of the thigh to detect stepping [32]. The device measures number of sit to stand transitions by monitoring changes in posture (e.g., moving from sitting/lying to standing and vice versa). The device samples at 20 Hz with a recording interval of 0.05 seconds. The activPAL has been shown to provide valid estimates of sedentary time in adults and children [33],[34],[36].

### 2.4. Processing ActivPAL Data

Raw activPAL data were processed using activPAL software. Using the “summary by week” file generated from the activPAL software, data were summarised by minute and reported as a proportion for each hour of the day, which was then summed to obtain time (in hrs/day) spent sitting/lying, standing and stepping for each 24 hour monitored period. As participants were not required to remove the activPAL whilst sleeping (or during washing/bathing/swimming), sleep time (recorded in a diary and verified from data recorded by the device) was removed from the activPAL summary data. Total waking hours spent sitting/lying, standing and stepping was summed for each monitored day. To be included in the data analysis, participants had to wear the activPAL monitor for at least 6 waking hrs/day for at least 3 days [37] (participants who did not provide valid weekend days were included in the data analysis for overall and week days,see data analysis section for further information). Since compliance with activity monitoring tends to be lower for young children [36], preschool children were included in the analysis if they wore the monitor for at least 6 waking hrs/day for at least 2 days. The following variables were computed: total waking time spent sedentary (in hrs/day, average of all valid days), % of waking day spent sedentary (average of all valid days) and total number of breaks in sedentary time per day (average of all valid days). These variables were also calculated for an average week day and average weekend day (for participants who provided valid weekend days).

### 2.5. Data Analysis

Associations between parents and children for sedentary time and number of breaks in sedentary time for overall (i.e. average of all valid days), week days and weekend days were assessed by Pearson correlations. Participants who did not provide a valid weekend day were included in the data analysis for overall and weekdays. Differences in sedentary time and number of breaks in sedentary time between weekdays and weekend days for each child group was assessed using t tests (children who did not provide a valid weekend day were excluded from this analysis). Statistical significance was set at *p* < 0.05 and Cohen's effect size for the correlation (>0.1: small; >0.3: medium and >0.5: large) was used to interpret the meaningfulness of correlation coefficients. All statistical procedures were conducted using SPSS version 22.

## 3. Results

Characteristics of the three parent-child dyad groups are presented in Table 1.

**Table 1.** Characteristics of the three parent-child dyad groups.

	N	M/F	Age (yrs)	Height (cm)	Body Mass (kg)	BMI (kg/m^2^)
**Preschool**						
Child	16	10/6	3(1)	98(9)	15.9(4.1)	16.7(2.5)
Parent	16	2/14	33(6)	166(7)	67.9(11.1)	24.6(3.6)
**Primary**						
Child	16	7/9	8(2)	127(16)	29.5(7.2)	18.1(1.7)
Parent	16	9/7	38(5)	169(10)	72.1(11.6)	25.3(3.9)
**Secondary**						
Child	19	12/7	14(2)	164(10)	55.7(12.3)	20.4(3.4)
Parent	19	5/14	45(7)	169(7)	71.9(15.3)	25.1(5)

Data reported as mean (SD); Abbreviations: N: number; M/F: Male/Female; BMI: Body Mass Index; cm: centimeters; kg: kilograms.

### 3.1. Descriptive Data for Sedentary Time and Sedentary Breaks

All parents and children of primary and secondary school age provided at least 4 valid days of data (see Table 2). In addition, the average waking wear time was >11 hours/day, which greatly exceeded our minimum wear time criteria (i.e. at least 6 hours/day for ≥3 days). In the preschool group, 7 children provided 2 valid days of data and the remaining children (n = 9) provided 3 or more valid days; the average waking wear time was >11 hours/day, which again exceeded our minimum wear time criteria (i.e. at least 6 waking hrs/day for ≥2 days).

#### 3.1.1. Overall Sedentary Time and Sedentary Breaks

Descriptive data for overall sedentary time and number of sedentary breaks are displayed in Table 2. Secondary school aged children spent a greater amount of time sedentary, greater percentage of their day sedentary, and had fewer breaks in sedentary time than preschool and primary school aged children.

#### 3.1.2. Weekday and Weekend Sedentary Time and Sedentary Breaks

There were no differences between week- and weekend- days for any of the variables for the preschool, primary or secondary groups (all *p* > 0.05). In primary school aged children, the percentage of waking day sedentary was greater at the weekend compared to weekdays (Table 2) however did not reach statistical significance (*p* = 0.086, 95% CI–1.2% to 16.6 %).

### 3.2. Parent and Child Associations for Sedentary Time and Sedentary Breaks

#### 3.2.1. Overall Sedentary Time and Sedentary Breaks

There were no significant associations between preschool children and their parents and secondary school children and their parents for time sedentary (preschool: *r* = 0.048, *p* = 0.859; secondary: *r* = −0.167, *p* = 0.494), or percentage of waking day sedentary (preschool: *r* = −0.208, *p* = 0.439; secondary: *r* = −0.247, *p* = 0.308). For the primary school parent dyad, there were medium, but non-significant positive correlations for time sedentary (*r* = 0.38, *p* = 0.146) and percentage of day sedentary (*r* = 0.363, *p* = 0.167).Preschool children and their parents reported a medium effect for the number of sedentary breaks, which approached statistical significance (*r* = 0.485, *p* = 0.057). This correlation was not observed in either of the other two groups (primary: *r* = 0.071, *p* = 0.793; secondary: *r* = 0.029, *p* = 0.906).

#### 3.2.2. Weekday and Weekend Sedentary Time and Sedentary Breaks

During weekend days the preschool child/parent dyad reported a large and significant positive association for number of sedentary breaks (*r* = 0.648, *p* = 0.012) but not during weekdays (*r* = −0.392, *p* = 0.133). The preschool child/parent dyad reported a medium effect for the percentage of waking day being sedentary at weekends (*r* = 0.479, *p* = 0.083, *r*^2^ = 0.23), however this did not reach statistical significance. There was no effect on this outcome during weekdays (*r* = 0.040, *p* = 0.884). There were no other significant associations (all *p* > 0.1) for the dyad groups during weekday and weekend days, with all groups reporting similar correlations for all variables to the overall data.

**Table 2.** Descriptive data for time spent sedentary and number of sedentary breaks in children and parents.

	Preschool		Primary		Secondary	
	Child	Adult	Child	Adult	Child	Adult
**No Valid Days**	3 (1)	4 (1)	5 (2)	6 (2)	6 (1)	6 (1)
**Wear Time (hrs/day)**	11.74 (1.41)	13.81 (1.71)	11.67 (2.01)	13.69 (1.90)	13.45 (1.12)	14.61 (1.32)
***Overall***	*n = 16*		*n = 16*		*n = 19*	
**Time Sedentary (hrs/day)**	6.70 (1.52)	7.58 (1.13)	6.68 (1.33)	7.77 (1.98)	8.69 (1.35)	8.57 (1.52)
**Time Sedentary (% of day)**	57.4 (7.3)	56.8 (9.2)	57.3 (5.0)	56.6 (11.3)	64.5 (8.5)	60.3 (11.5)
**Number of Sedentary Breaks**	130 (47)	65 (22)	100 (33)	61 (20)	54 (14)	55 (18)
***Weekday***	*n = 9*		*n = 14*		*n = 15*	
**Time Sedentary (hrs/day)**	7.12 (2.57)	7.95 (1.82)	6.21 (2.27)	7.78 (2.24)	8.90 (1.23)	8.60 (1.80)
**Time Sedentary (% of day)**	55.6 (11.3)	51.9 (11.4)	51.4 (15.9)	55.9 (14.4)	63.8 (8.0)	57.4 (12.0)
**Number of Sedentary Breaks**	147 (65)	76 (23)	100 (36)	62 (26)	58 (14)	63 (23)
***Weekend***	*n = 9*		*n = 14*		*n = 15*	
**Time Sedentary (hrs/day)**	5.82 (3.31)	7.03 (2.45)	7.00 (1.70)	8.23 (1.71)	7.83 (1.92)	8.29 (2.27)
**Time Sedentary (% of day)**	54.7 (21.6)	53.8 (9.5)	59.2 (5.5)	56.6 (10.4)	61.9 (11.8)	61.3 (15.0)
**Number of Sedentary Breaks**	127 (73)	65 (27)	98 (38)	64 (21)	55 (17)	28 (13)

Data presented as mean (standard deviation).

## 4. Discussion

Results from the present study show that children of all ages spent a large proportion of their waking day sedentary. Time spent sedentary was similar in preschool (6.7 hours/day, 57.4% of waking day) and primary school aged children (6.7 hours/day, 57.3% of waking day) and was higher among secondary school aged children (8.7 hours/day, 64.5% of waking day). In addition, sedentary time was high during weekdays and weekend days in each child group, with no marked differences between weekdays and weekends. Consistent with our findings, several studies have reported that objectively measured sedentary time is high in children and increases with age [2]–[4]. However, it is difficult to directly compare our results with previous research due to differences in samples and age ranges of the children studied, as well as different devices used to objectively measure sedentary time. Most studies have used the ActiGraph accelerometer to objectively measure sedentary time in young people [2]–[4], whereas very few studies have used the activPAL, which provides a more accurate estimate of sedentary time compared to accelerometer-based devices [33],[34]. The present study has provided robust estimates of sedentary time in a small sample of young children, primary and secondary school aged children and shows that sedentary time is high in all three groups, especially adolescents, both during weekdays and weekend days. Therefore, interventions to reduce sedentary time are needed for young people of all ages and should target both weekdays (e.g. the school setting and after school period) as well as weekends.

The primary aim of the present study was to examine associations between parent and child activPAL measured sedentary time and the number of breaks in sedentary time on weekdays and weekend days across three age groups of children (i.e. pre-school children age 2–4 years, primary school children aged 5–11 years and adolescents aged 12–17 years). Results showed that associations in activPAL measured sedentary time between parents and children ranged from null to medium, and varied by age of the child and by time of the week. For the secondary school-parent dyad, there were no significant associations for overall sedentary time or for weekday and weekend sedentary time. It is possible that adolescents spend less time with their parents compared to younger children and therefore may be less likely to be influenced by their parents' sedentary time. In the primary school-parent dyad, we observed medium positive correlations, though not statistically significant, for overall sedentary time (*r* = 0.38) and percentage of waking day sedentary (*r* = 0.36). These findings suggest that primary school children may be more likely to be sedentary if their parents spend a lot of time being sedentary, though it is difficult to draw any firm conclusions due to the small sample size and cross-sectional study design. Similar to our findings for the parent-primary school dyad, Jago et al [30] reported small positive correlations (*r* = 0.18–0.19, *p* < 0.05) in ActiGraph measured sedentary time between parents and their daughters (aged 10–11 years) and sons (aged 10–11 years). Jago et al [30] also found that only 12% of children's sedentary time was explained by parental sedentary time, suggesting that other factors may have a stronger influence on children's sedentary time. Fuemmeler et al [31] examined associations in ActiGraph measured sedentary time between parents and 10 year old children by time of day and week, and by gender of children and parents. The only significant association was between fathers and their children on weekend days, whereas sedentary time of parents and children of both genders were not correlated on weekdays, during the after-school period or for mothers and their children on weekend days. In contrast to these findings by Fuemmeler et al [31], we found similar correlations (i.e. medium positive) for overall, weekday and weekend sedentary time for the primary school-parent dyad. However, we did not examine the association in sedentary time during the after-school period and the number of parents and children in each age group was too small to explore associations by gender. These should be explored in future studies using activPAL monitors.

It is possible that the association between parent-child sedentary time may be stronger for young children as they are likely to spend more time with their parents compared with older children and adolescents. In the present study, there was no significant correlation between parents and young children for overall sedentary time or percentage of waking day sedentary, however there was a medium, positive correlation, though not statistically significant, for percentage of waking day sedentary at the weekend (*r* = 0.48). Further analysis showed that a small proportion (23%) of preschool children's sedentary time at the weekend was explained by their parents' weekend sedentary time. The association between parent-preschool child sedentary time may be stronger on weekend days because parents may spend more time with their children on weekends and may have more influence on their young child's sedentary behaviours during this time period. A recent study [32] found positive associations for objectively measured sedentary time in mothers and their 4 year old children, with the strongest associations occurring in the morning (6 am–12 pm) compared to afternoon (12–5 pm) and evening (5–11 pm), suggesting that the influence of mother's sedentary behaviour on their young child's sedentary behaviour differs by time of day. Although we did not specifically examine the mother-child association, most (88%) of the parents who participated in the present study were mothers.

In the parent-preschool dyad, we observed a significant, large correlation in the number of breaks in sedentary time, particularly at the weekend (*r* = 0.65), whereas there were no associations for frequency of sedentary breaks between primary and secondary school children and their parents. The number of sedentary breaks was higher in preschool children (130/day) compared to primary (100/day) and secondary (54/day) school children, which can be explained by the more sporadic nature of younger children's movements. Although we observed a significant association in the frequency of sedentary breaks in the preschool-parent dyad, it is difficult to determine the direction of the association (i.e., is the young child's behaviour influencing their parent's number of sedentary breaks or is the parent's behaviour influencing their young child's number of sedentary breaks?). Thus, future research should explore the nature of this relationship in more detail and the influence this may have on the health of young children and their parents. Furthermore, the number of sedentary breaks was much lower in secondary school children compared to younger children (and was similar to the parent group), suggesting that secondary school children are more at risk of prolonged periods of sedentary time, thus interventions should promote breaks in sedentary time as well as reducing total sedentary time in this age group.

To our knowledge, this is the first study to examine associations in objectively measured sedentary time and frequency of sedentary breaks between parents and children from three different age groups using an activPAL posture sensor, which provides a more accurate estimate of sedentary time compared to accelerometer-based motion sensors. A small number of studies, discussed above [30]–[32], have examined parent-child associations using accelerometer-based motion sensors to measure sedentary time in children and their parents and have reported mixed associations. The inconsistent findings (between our study and the accelerometer-based studies as well as among the accelerometer based studies themselves) may be due to variation in samples and age ranges of the children studied, different types of devices to measure sedentary time and different methods to process the output from these devices. However, there is some evidence from the present study and from the accelerometer-based studies to suggest that parent-child associations in sedentary time varies by age of the child, gender of the child and of the parent, and by time of day and time of the week. Limitations of this study include the cross sectional design and a small sample of parents (mainly mothers), children and adolescents from one geographical area that may not be representative of all populations.

## 5. Conclusions, Implications and Future Research

Overall, the present study found no significant associations in activPAL measured sedentary time between secondary school aged children and their parents, however due to the high levels of sedentary behaviour in adolescents, it is important to identify the factors that may have a strong influence on objectively measured sedentary time in this age group so that these correlates can be targeted in interventions. Furthermore, secondary school children were more sedentary and had fewer sedentary breaks than younger children, thus interventions should promote breaks in sedentary time as well as reducing total sedentary time in this age group. There were medium positive correlations in activPAL measured sedentary time between primary school children and their parents, and between preschool children and their parents at the weekend. Therefore, interventions aimed at reducing sedentary behaviour of parents and children together, particularly at the weekend for young children, may be effective in these age groups. However, since the size of the associations are not substantial (i.e. small–medium), other factors may have a stronger influence on children's sedentary time and should be identified and targeted in sedentary behaviour interventions. Despite these findings, conclusions from this study should be taken with some caution due to the small sample size and cross-sectional study design. Future research using the activPAL posture sensor is therefore required in order to provide robust estimates of sedentary time in large samples of parents and children and to further explore parent-child associations by age of the child, gender of the child and parent, and by time of day and time of the week, to better understand the extent to which parent's sedentary time influences their children's sedentary time.
